# Type I interferon signaling, cognition and neurodegeneration following COVID-19: update on a mechanistic pathogenetic model with implications for Alzheimer’s disease

**DOI:** 10.3389/fnhum.2024.1352118

**Published:** 2024-03-18

**Authors:** George D. Vavougios, Vasilis-Spyridon Tseriotis, Andreas Liampas, Theodore Mavridis, Gabriel A. de Erausquin, Georgios Hadjigeorgiou

**Affiliations:** ^1^Department of Neurology, Medical School, University of Cyprus, Lefkosia, Cyprus; ^2^Laboratory of Clinical Pharmacology, Aristotle University of Thessaloniki, Thessaloniki, Greece; ^3^Tallaght University Hospital (TUH)/The Adelaide and Meath Hospital Dublin, Incorporating the National Children's Hospital (AMNCH), Dublin, Ireland; ^4^Laboratory of Brain Development, Modulation and Repair, The Glenn Biggs Institute of Alzheimer's and Neurodegenerative Disorders, Joe R. and Teresa Lozano Long School of Medicine, The University of Texas Health Science Center at San Antonio, San Antonio, TX, United States

**Keywords:** Alzheimer’s disease, COVID-19, interferons, drug repositioning, innate immunity, neurogenesis, cognitive impairment

## Abstract

COVID-19’s effects on the human brain reveal a multifactorial impact on cognition and the potential to inflict lasting neuronal damage. Type I interferon signaling, a pathway that represents our defense against pathogens, is primarily affected by COVID-19. Type I interferon signaling, however, is known to mediate cognitive dysfunction upon its dysregulation following synaptopathy, microgliosis and neuronal damage. In previous studies, we proposed a model of outside-in dysregulation of tonic IFN-I signaling in the brain following a COVID-19. This disruption would be mediated by the crosstalk between central and peripheral immunity, and could potentially establish feed-forward IFN-I dysregulation leading to neuroinflammation and potentially, neurodegeneration. We proposed that for the CNS, the second-order mediators would be intrinsic disease-associated molecular patterns (DAMPs) such as proteopathic seeds, without the requirement of neuroinvasion to sustain inflammation. Selective vulnerability of neurogenesis sites to IFN-I dysregulation would then lead to clinical manifestations such as anosmia and cognitive impairment. Since the inception of our model at the beginning of the pandemic, a growing body of studies has provided further evidence for the effects of SARS-CoV-2 infection on the human CNS and cognition. Several preclinical and clinical studies have displayed IFN-I dysregulation and tauopathy in gene expression and neuropathological data in new cases, correspondingly. Furthermore, neurodegeneration identified with a predilection for the extended olfactory network furthermore supports the neuroanatomical concept of our model, and its independence from fulminant neuroinvasion and encephalitis as a cause of CNS damage. In this perspective, we summarize the data on IFN-I as a plausible mechanism of cognitive impairment in this setting, and its potential contribution to Alzheimer’s disease and its interplay with COVID-19.

## 1 Background

Our current understanding of COVID-19 is that long term consequences may arise following the acute phase, even in milder cases. Collectively, these syndromes and symptoms exist within what is defined as the long COVID or post-acute COVID-19 condition (PASC) spectrum (Parotto et al., 2023). Of note, neuropsychiatric manifestations and specifically cognitive decline are consistently reported in a subset of long COVID patients (Deer et al., 2021). In 2022, a joint international task force by WHO and Alzheimer’s Association reported on a protocol to systematically study the neuropsychiatric sequalae of COVID-19, and provided a comprehensive review of the evidence linking these manifestations with Alzheimer’s disease and related dementias (ADRD). In that protocol, we specifically proposed that SARS-CoV-2 would trigger ADRD-like pathology following the extended olfactory cortical network (EOCN) in older individuals with underlying genetic susceptibility (de Erausquin et al., 2021, 2022). This hypothesis emerged from the descriptions of anosmia as and early and often single symptom of SARS-CoV-2 infection, and the well-known association of the same symptom with cognitive impairment in Alzheimer’s disease (Roberts et al., 2016; Tian et al., 2022; Xiao et al., 2023).

Persistent hyposmia has been correlated with cognitive impairment in those previously exposed to COVID-19, with functional MRI (fMRI) revealing altered olfactory network connectivity in the absence of structural damage (Muccioli et al., 2023). In FDG PET studies of post-acute COVID-19 patients, hypometabolism involving areas involved in olfactory and limbic pathways has also been associated with corresponding symptoms (Verger et al., 2022). Parallels between early Alzheimer’s disease findings and COVID-19 associated neuronal damage can also be seen by the UK Biobank study, where both diffuse grey matter loss as well as focal to the EOCN and the parahippocampal/perirhinal and entorhinal cortex was identified in COVID-19 patients (Douaud et al., 2022). The implication of the EOCN and cognition is especially relevant to COVID-19, as it one sites SARS-CoV-2 either uses to port in the CNS, or cause distal inflammation communicated to proximal sites in a sterile manner. This neuroanatomical concept has been supported by emerging neuropathological studies on both animal models (Jiao et al., 2021; Philippens et al., 2022) and via human brain autopsy findings (Thakur et al., 2021).

In rhesus monkeys, SARS-CoV-2’s olfactory neuroinvasion route was shown to result gliosis and neuronal apoptosis involving the entorhinal area, hippocampus, thalamus, and midbrain in differing degrees (Jiao et al., 2021). Similarly, in a Syrian golden hamster model of SARS-CoV-2 transolfactory neuroinvasion, the hippocampi displayed decreased density of dendritic spines tandem with gliosis in the olfactory bulb (Kishimoto-Urata et al., 2022). Murine models currently indicate that both microgliosis and proteinopathy may persist beyond the acute phase of COVID-19 (Käufer et al., 2022; Kishimoto-Urata et al., 2022). As we have previously expanded on, microgliosis, tauopathy and Alzheimer’s disease-like signaling is consistently shown in brain organoids exposed to SARS-CoV-2 and mediated by IFN-I (Ramani et al., 2020; Hou et al., 2022; Kong et al., 2022, 2023), whereas this model in humans is supported by radiological evidence and neuropathology that parallels Alzheimer’s disease in its early stages, involving olfactory and limbic networks (de Erausquin et al., 2022; Douaud et al., 2022; Reiken et al., 2022; Ferrucci et al., 2023).

Similar findings have been revealed by human autopsy studies. In a study of consecutive COVID-19 patients (*n* = 41), microgliosis, reactive astrocytosis and neuronophagia were consistent findings, localized in the olfactory bulb, brainstem, and thalami of a subset of patients, along with ischemic / hypoxic lesions. Notably, autopsied brains contained neurofibrillary tangles, with and without amyloid plaques (17/41, 41.4%), a finding disproportionate with the age of the patients (Thakur et al., 2021). Microgliosis localized to the hippocampus and the brainstem has been consistently reported in other human neuropathological studies of COVID-19 patients, albeit in the absence of productive SARS-CoV-2 replication within the CNS (Lou et al., 2021; Poloni et al., 2021).

These observations have been reinforced by neuropathological findings from brain and brain plus fused cortical-blood vessel organoid models of SARS-CoV-2 infection, where SARS-CoV-2 infected neurons displayed aberrant tau phosphorylation and localization (Ramani et al., 2020). In the fused cortical blood vessel organoid model, tauopathy, beta amyloidosis and microglial activation was also noted as a result of SARS-CoV-2 infection (Kong et al., 2023). Notably, aberrant tau phosphorylation secondary to SARS-CoV-2 infection has also been observed in SH-SY5Y neuroblastoma cells (Di Primio et al., 2023). Type I interferon responses were elicited by SARS-CoV-2 infection of forebrain organoids, potentially in variant-dependent manner (Hou et al., 2022). IFN-I production by SARS-CoV-2-infected astrocytes in an organoid model, mediated by neuropillin-1. IFN-I signaling by infected astrocytes was shown to lead to decreased neurotransmitter levels, including choline, and neuronal destruction (Kong et al., 2022). Even in the absence of productive replication, IFN-I responses, microgliosis and a transcriptomic profile resembling neurodegenerative disease has also been shown in another brain organoid model (Suzzi et al., 2023).

We proposed that similar molecular mechanisms overlap between Alzheimer’s disease (AD) and COVID-19, with a focus on Type I interferon signaling (IFN-I) (Vavougios et al., 2022a). There are several reasons why IFN-I may account for the effects of COVID-19 on the CNS, and their overlap with Alzheimer’s disease pathogenesis. Type I interferon signaling is the premier response to viral infection, regardless of pathogens involved. This tonic innate immune signal is ubiquitous in the periphery as well as the CNS and remains in a prolonged stimulation determined by the host’s immune fitness and microbiome immunosurveillance (Abt et al., 2012; Lukhele et al., 2019; Wu et al., 2022). Peripheral infection can affect central sites such as the hippocampi in a sterile manner, and such prolonged stimulation may affect hippocampal neurogenesis and plasticity via crosstalk with the resident homeostatic IFN-I (Ekdahl et al., 2003; Michalovicz et al., 2015; Hosseini et al., 2020; Petrisko et al., 2020; Bayat et al., 2022). Type I interferon signaling has been shown to both directly affect cognition, and modulate long-term neuroinflammatory and neurotoxic effects for Alzheimer’s disease through microgliosis (Roy et al., 2020, 2022), beta-amyloidosis (Hur et al., 2020) and tauopathy (Jin et al., 2021; Udeochu et al., 2023). A growing body of human studies and disease models of Alzheimer’s disease consistently reports on dysregulated type I interferon signaling (See Supplementary Material 1 for an extended presentation of relevant studies) (Blank and Prinz, 2017; Jin et al., 2022; Sanford and McEwan, 2022; Vavougios et al., 2022b).

In COVID-19, type I interferon signaling is a primary innate immune response against SARS-CoV-2 that has been shown to be dysregulated as a result of complex host-response interactions (Lee and Shin, 2020; Shi et al., 2021; Eskandarian Boroujeni et al., 2022; Wu et al., 2022). Dysregulated type I interferon signaling has been shown to extend to sites proximal to the CNS, such as the olfactory bulb, the choroid plexus, and brain endothelial cells (reviewed in Vavougios et al., 2022a; Crook et al., 2023). Within the context of the studies presented, this peripheral activation of IFN-I due to COVID-19 can affect the CNS with a predilection for neurogenesis sites such as the hippocampus and the olfactory bulb (Ekdahl et al., 2003; Baruch et al., 2014; Bayat et al., 2022; Stępień et al., 2023), correspondingly mediate cognitive impairment (Blank et al., 2016) and anosmia (Llana et al., 2022, 2023).

## 2 Tonic type I interferon signaling dysregulation as a shared mechanism between COVID-19 and Alzheimer’s disease

Previous works from our group developed a model for the pathogenesis of CNS injury following COVID-19, and the overlap between the mechanisms that mediated with Alzheimer’s disease (Vavougios et al., 2021b, 2022a,b,c). We built this model upon *in silico* evidence of overlapping immune pathway dysregulation between peripheral blood and central nervous system sites in Alzheimer’s disease patients (Vavougios et al., 2020). Considering the established roles of IFN-I in cognition, immunity, and the pathogenesis of Alzheimer’s disease (Roy et al., 2020; Jin et al., 2022; Sanford and McEwan, 2022; Song et al., 2022), it is reasonable to posit that a pathogen that directly challenges IFN-I such as SARS-CoV-2 is an adequate probe into IFN-I mediated neuroinflammation and neurodegeneration.

What our model specifically describes is the transmission of an outside-in, quasi-infectious type I interferon signal from the periphery to the CNS, potentially via extracellular vesicles, which results in the disruption of tonic IFN-I centrally. Quasi-infectious here is used to describe the initial stimulation of IFN-I signaling by a pathogen, and the subsequent sterile elicitation of IFN-I activation centrally by endogenous disease associated molecular patterns (DAMPs). This transmission would require the interface between a peripheral site (e.g., the olfactory neuroepithelium, the brain vascular endothelium; Vavougios et al., 2022c) and a central site (such as the hippocampus) (Vavougios et al., 2021b). While peripheral stimulation persists, central sites would be exposed to prolonged IFN-I signaling and proinflammatory conditions which would be deleterious to neurogenesis, synaptogenesis and by extent, cognition (Vavougios et al., 2022a,b; Crook et al., 2023). Once IFN-I signaling has been localized to the CNS, it may become feed-forward due to the production of (DAMPs), a concept displayed experimentally in Alzheimer’s disease (Roy et al., 2020, 2022) with therapeutic implications for targeting both microglial responses and IFN-I (Moore et al., 2020). To probe into IFN-I dysregulation, we had focused on the interferon induced transmembrane protein 3 (IFITM3) (Vavougios et al., 2021b), an innate immunity protein effective against multiple pathogens, including SARS-CoV-2 (Prelli Bozzo et al., 2021) and a modulator of beta-amyloidogenesis, with an emerging role in the pathogenesis of Alzheimer’s disease (Hur et al., 2020). We had furthermore hypothesized that exosomal tau, shown to be closely related to beta amyloid and capable of seeding (Miyoshi et al., 2021; Polanco and Götz, 2022) would also intrinsically enhance feed-forward activation of IFN-I in the CNS. Insoluble tau uptake has been previously demonstrated in microglia (Bolós et al., 2016), and via exosome synthesis, mediate seeding (Asai et al., 2015). A subset of our hypothesis has been recently demonstrated by the pathogenic tau-mediated activation of IFN-I and cyclic GMP–AMP synthase (cGAS) in microglia, leading to diminished cognitive resilience in a murine model (Udeochu et al., 2023). Our model does neither rely on, or contradict neuroinvasion; instead, transolfactory neuroinflammation would result in, e.g., a direct elicitation of IFN-I in the hippocampus via EOCN projections (de Erausquin et al., 2022; Vavougios et al., 2022a).

Following the formulation of these works and their publication, a growing body of studies has provided robust corroboration to the mechanisms we predicted.

In this perspective, we aim to present our model within the context these studies, and the potential implications for a type I interferon-centric model for the pathogenesis of Alzheimer’s disease and post-acute COVID-19 cognitive impairment with implications for their treatment.

## 3 Type I interferon signaling dysregulation in the brain is a mechanistic cause of neuronal damage and neuroinflammation in COVID-19

In the central nervous system, type I interferon responses represent an innate immune mechanism that is responsive to peripheral inflammation, and maintains the balance between danger-associated molecular pattern (DAMP) recognition and the magnitude of the inflammatory response that will sufficiently lead to the amelioration of this initial immune stimulant (Blank and Prinz, 2017). Homeostatic IFN-I responses in the human brain decrease with increased age, favoring inflammation (Baruch et al., 2014). In this setting, cognitive impairment has been shown to occur both as consequence of IFNAR activation (McNab et al., 2015), via a relatively indolent synaptopathy due to the chronic accumulation and dissemination of neurotoxic molecules and proteopathic seeds (Baruch et al., 2014; Viengkhou and Hofer, 2023).

We have previously adapted this well-observed paradigm to formulate the hypothesis of an outside-in injury of the CNS during COVID-19, where immune crosstalk results in microglial activation and immune-mediate neuronal injury (Vavougios et al., 2022a). In our model, IFN-I dysregulation occurs in the setting of peripheral infection and is communicated centrally via sites such as the olfactory bulb, the brainstem nuclei and the blood–brain barrier (Meinhardt et al., 2021; Krasemann et al., 2022). Sites specifically vulnerable to dysregulated IFN-I such as the olfactory bulb and the hippocampus (Bayat et al., 2022; Stępień et al., 2023) may furthermore readily account for both clinical (Gonzalez-Aleman et al., 2022) and neuropathological (Lou et al., 2021) manifestations of the CNS involvement in COVID-19.

The generation of amyloid-beta and hyperphosphorylated tau, molecules that we currently recognize as inducers of innate immunity (Rexach et al., 2020; Roy et al., 2020; Jin et al., 2021; Roy et al., 2022; Vavougios et al., 2022a; Udeochu et al., 2023), can in turn sustain pathogenic IFN-I in a feed-forward manner acting as danger associated molecular pattern (Asai et al., 2015; Bolós et al., 2016) in a process that has been shown to prime microglia towards phenotypes specifically associated with the earlier stages of neurodegenerative disease (Roy et al., 2020; Jin et al., 2021; Magusali et al., 2021; Kim et al., 2022; Udeochu et al., 2023). This aspect of our model, previously predicting beta amyloidosis and tauopathy based on the IFN-I response secondary to SARS-CoV-2 infection (Ramani et al., 2020; Vavougios et al., 2021a,b, 2022a,b,c; Di Primio et al., 2023) has been independently validated (Ramani et al., 2020; Green et al., 2022; Käufer et al., 2022; Ma et al., 2022; Reiken et al., 2022; Di Primio et al., 2023; Suzzi et al., 2023). A notable part of this feed-forward process is that microglial activation may be sustained via sterile DAMPs as IFN-I activators (Roy et al., 2020) such as exosomal or insoluble tau (Asai et al., 2015; Bolós et al., 2016) and errant nucleic acids (Roy et al., 2020). Molecular markers of neuronal damage have been identified in COVID-19 patients (Frontera et al., 2022; Lennol et al., 2023), albeit they are currently not routinely used along the main molecular assays used to detect COVID-19 (Habibzadeh et al., 2021).

## 4 Are SARS-CoV-2-introduced perturbations in type I interferon signaling a plausible mechanism for the observed effects on cognition?

Our model posits that the induction of peripheral IFN-I during COVID-19 may dysregulate IFN-I homeostasis in the CNS. A consequent question is whether these dysregulations are a valid biological substrate for cognitive impairment.

IFN-I represents a canonical immune surveillance response for the brain that effectively regulates pathogen dynamics and cellular permissiveness (Drokhlyansky et al., 2017; Welsch Jeremy et al., 2019). The relationship between IFN-I and cognition relies on data from several translational models that indicate its primary homeostatic role in the form of a tonic IFN-I response that oscillates between inflammation and quiescence. Homeostatic roles for type I interferon have been described in the maintenance of hippocampal synaptic plasticity by astrocytes (Hosseini et al., 2020). Tissue specific activation of the interferon alpha and beta receptor subunit 1 (*IFNAR1*) and the C-X-C Motif Chemokine Ligand 10 - C-X-C Motif Chemokine Receptor 3 (*CXLC10-CXCR3*) axis in murine brain endothelial and epithelial cells may manifest as cognitive impairment (Blank et al., 2016). Furthermore, they showed that this induction may occur in the setting of immune challenge from either viral ssRNA or type I interferons. As an ssRNA virus, SARS-CoV-2 has been shown to productively infect brain endothelial cells and alter IFN-I signaling via host-virus protein–protein interactions (Wenzel et al., 2021; Krasemann et al., 2022; Vavougios et al., 2022c; Yang et al., 2022; Suzzi et al., 2023). such as *Nsp5* mediated cleavage of nuclear factor (NF)-κB essential modulator (NEMO) (Wenzel et al., 2021). Interferon signaling is also spatially resolved, however. Sites of adult neoneurogenesis have been shown to be more vulnerable to aberrant IFN-I signaling, including the subventricular zone (SVZ) and the hippocampus (Blank and Prinz, 2017; Viengkhou and Hofer, 2023) – sites furthermore important in the setting of Alzheimer’s disease (Kim et al., 2022), with the EOCN potentially affected in early or preclinical stages (Roberts et al., 2016; Yahiaoui-Doktor et al., 2019; Tian et al., 2022; Xiao et al., 2023). This concept appears to account for this specific aspect of COVID-19 neuropathology, where microgliosis (Poloni et al., 2021) and a decrease in adult human neoneurogenesis are combined (Stępień et al., 2023). Notably, aside from disease-associated microglia (DAM), IFN-I expressing microglia have also been shown to enhance neurodegeneration as well as the infiltration of the SVZ by T helper cells (Zhang et al., 2023). The interaction between microglia and T cells in the SVZ are state specific, with an apparent infiltration occurring with advanced age (Kim et al., 2022), when basal IFN-I responses are also altered (Baruch et al., 2014). Excessive exposure to the secretion of type I interferon by the microglia either *in vitro* or *in vivo* can lead to derangement of functional neuronal maturation (Hattori et al., 2020). Notably, tau and beta amyloid have been shown to stimulate microglia and upregulate IFN-I via cGAS-STING, a pathway that is furthermore linked to IFITM3 (Jin et al., 2021; Wu et al., 2023) further linking innate immunity and Alzheimer’s disease.

## 5 Type I interferon signaling as a plausible mechanism for both COVID-19-associated cognitive impairment and Alzheimer’s disease, and its implications for pathogenesis and treatment

So far, we have established a robust body of works that validate our hypothesis of IFN-I as a central hub for cognitive impairment and neuronal damage in the setting of COVID-19. The convergence on IFN-I however constitutes a central part of the molecular, transcriptional and neuroanatomical overlap between COVID-19 and neurodegenerative disease, and specifically with Alzheimer’s disease (Magusali et al., 2021; Bayat et al., 2022; Mavrikaki et al., 2022; Vavougios et al., 2022a,b,c; Suzzi et al., 2023).

Similarly, a growing body of studies identify IFN-I responses as key molecular evens in the evolution of Alzheimer’s disease (Taylor et al., 2014; Roy et al., 2020; Sanford and McEwan, 2022; Govindarajulu et al., 2023; Sanford et al., 2023; Udeochu et al., 2023). Several works localize relationship in microglial responses to IFN-I, and their potential to drive feed-forward amyloidogenic cascades (Moore et al., 2020; Roy et al., 2020) and mediate exosomal tau seeding (Bolós et al., 2016; Wang et al., 2017; Miyoshi et al., 2021; Polanco and Götz, 2022; Udeochu et al., 2023). Aberrant IFN-I signaling in microglia has been shown to display tau-induced dystrophic and senescent phenotypes in murine models (Jin et al., 2022); recently, microglial activation in the setting of IFN-I signaling was shown to lead to synaptopathy and cognitive deficits, and conversely reversed by microglial *ifnar1* deletion (Roy et al., 2022). Another notable aspect of Type I interferon signaling perturbations in Alzheimer’s disease is that they are detectable both in peripheral immune cells and the CNS in Alzheimer’s disease (Roy et al., 2020; Song et al., 2022), a concept we have previously and independently outlined (Vavougios et al., 2020).

Within the context of our model, several specific conclusions regarding IFN-I, cognition and neurodegeneration can be made: (a) Peripheral perturbations in IFN-I signalling can be communicated to the CNS via multiple sites (b) dysregulation of constitutional IFN-I responses can manifest as cognitive impairment acutely, or as a result of cognitive impairment (c) Local IFN-I dysregulation can lead to microgliosis and proteinopathy (d) once dysregulated, IFN-I requires DAMPs but not necessary a proliferating pathogen in the CNS (e) Exosomal tau, neurofibrillary tangles and errant nucleic acids may act as such DAMPs (d) this model can be activated by multiple pathogens (Dominy et al., 2019) or commensal microbiota, (Abt et al., 2012; Supplementary Figure 3) as well as other conditions resulting in IFN-I dysregulation, such as hypoxia.

In this light, COVID-19 functions as a potent perturbator of IFN-I homeostasis, and via that dysregulation molecular mechanisms associated Alzheimer’s disease may be enhanced. This proposed concept is furthermore confirmed by the overlap we and others have observed between COVID-19 and Alzheimer’s disease, and the central role of interferon stimulated genes in the development of this pathology (Mostafavi et al., 2018; Magusali et al., 2021; Poloni et al., 2021; Vavougios et al., 2021a,b; Green et al., 2022; Reiken et al., 2022; Semerdzhiev et al., 2022; Suzzi et al., 2023). Furthermore, the dysregulation of tonic IFN-I rather than the specific effect of SARS-CoV-2 can generalize this model to other pathogens and infections that have been associated with increased susceptibility to Alzheimer’s disease (Levine et al., 2023).

## 6 Context and limitations

The interpretation of our model of cognitive impairment as well as the contribution of dysregulated tonic IFN-I and the crosstalk between peripheral and central nervous system immunity requires context and an awareness of important limitations (See Supplementary Material 2 for an extended presentation of context, strengths and limitations).

## 7 Conclusion

In this perspective, we presented the evolution of a model that explores how peripheral IFN-I dysregulation may affect the CNS, triggering microgliosis, proteinopathy and cognitive deficits. Furthermore, while COVID-19/PASC and Alzheimer’s disease are distinct conditions, our pathogenetic model provides insight into an implied intermediate mechanism by which infection, results in damage to structures affecting cognition and olfaction in the central nervous system, via IFN-I pathways that are also encountered in Alzheimer’s disease, and are druggable (Mavrikaki et al., 2022; Sanford and McEwan, 2022; Vavougios et al., 2022b). As innate immune- and specifically IFN-I dysregulation is increasingly recognized in Alzheimer’s disease, our model may be generalizable across pathogen and commensal microbiota-focused models where innate immunity is similarly stimulated (Dominy et al., 2019; Grabrucker et al., 2023; Levine et al., 2023).

**Figure 1:**
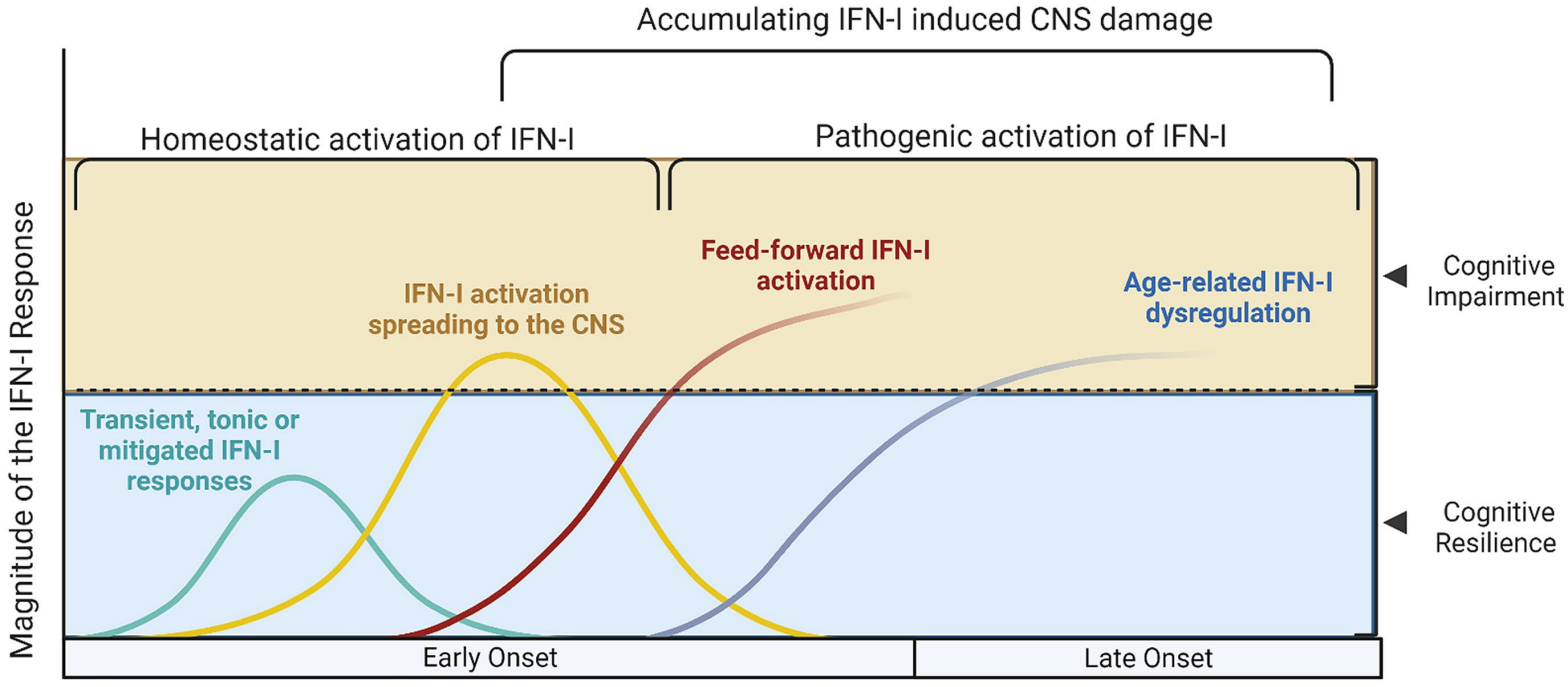
Different models of Type I interferon signaling in the human brain and their effects on cognition. Type I interferon signaling (IFN-I) is a primary innate immune response against pathogens and danger associated molecular patterns (DAMPs). A tonic response is maintained by commensal microbiota and transient immune challenges, whereas other, unmitigated sources of stimulation may exert a deleterious effect on the CNS. **(A)** The cyan curve represents a transient activation of IFN-I, where the source of DAMPs would be depleted, the offending pathogen dealt with and correspondingly, anti-inflammatory/homeostatic IFN-I responses would prevail., whereas in **(B)** the yellow curve represents hyperinflammatory IFN-I activation, which still however follows a canonical course and may be reversed. During this hyperactivation period, cognitive impairment may manifest, as is evidence from both murine and human studies; in this setting, acute COVID-19 and other infections would also fit. The red **(C)** and blue **(D)** lines represent cases where secondary, feed-forward activation of IFN-I responses by endogenous DAMPS exceeds the capacity of its regulatory mechanisms and may eventually establish the precipice of neurodegeneration. These curves are a function of the magnitude of IFN-I activation with age (early onset vs. late onset cognitive impairment and CNS damage, conversely).

**Figure 2:**
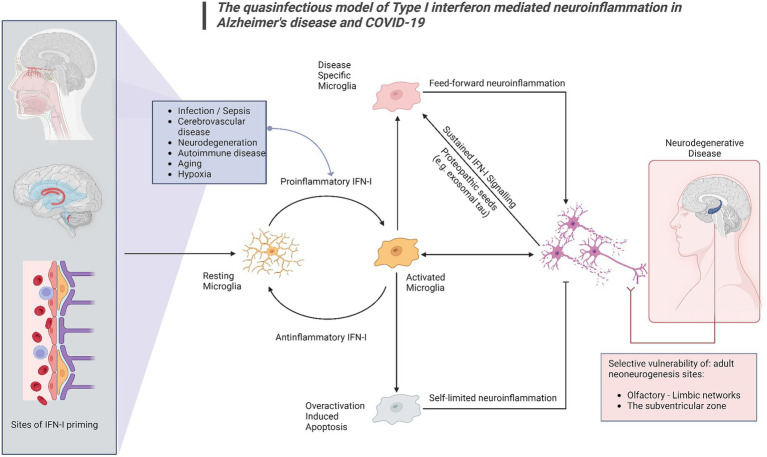
The quasinfectious model of Type I interferon mediated neuroinflammation in Alzheimer’s disease and COVID-19. In this model, applicable to both COVID-19-associated cognitive impairment and COVID-19, Proinflammatory IFN-I activation may occur distally via DAMPs and other IFN-I activated sites at the blood–brain barrier, the choroid plexus and the olfactory bulb. Prolonged infection, cerebrovascular disease, autoimmune disease, cancer, aging and pre-existing neurodegenerative disease may also generate endogenous DAMPs such as exosomal tau, nucleic acids and beta-amyloid. Microglia have been shown to react to these signals as IFN-I relays, switching from a resting to an activated state. A homeostatic response includes switching off proinflammatory IFN-I responses, extending to overactivation-induced apoptosis for microglia. A subsequent phenotypic switch in activated microglia, would subsequently promote neurodegeneration. Central nervous system sites adjacent to distal or peripheral inflamed sites would be primarily affected, as well as sites of adult neurogenesis, shown to be vulnerable in both COVID-19 and Alzheimer’s disease.

This proposed model provides mechanistic background for the combination of olfactory dysfunction and cognitive impairment, considering the selective vulnerability of the olfactory bulb-hippocampal circuitry to IFN-I as neurogenesis sites (Ekdahl et al., 2003; Bayat et al., 2022; Stępień et al., 2023; Figure 1). As shown by a growing body of research, IFN-I can furthermore account for the effect of age on inflammation and cognition (Baruch et al., 2014), which again is a central component in the pathogenesis of ADRD. Lastly, our model supports a multi- or sequential- hit concept where multiple pathogens and immune challenges to which IFN-I is responsive may result in lasting CNS damage (Figure 2). Following an initial pathogen-induced stimulation, endogenous DAMPs (such as nucleic acids and proteopathic seeds) produced by neuronal damage would fuel second-order IFN-I dysregulation by a sterile inflammatory response (Rubartelli, 2014). Notably, interferon stimulated genes such as IFITMs and OASs, as we and other have reported (Magusali et al., 2021; Vavougios et al., 2021a,b) are linked to nucleic acid surveillance mechanisms (Magusali et al., 2021; Vavougios et al., 2022b) such as the OAS antiviral response and cGAS-STING, where Αβ and tau pathobiology converge with IFN-I dysregulation and inflammasome activation (Magusali et al., 2021; Udeochu et al., 2023; Wu et al., 2023). Focusing on IFN-I modulation and restoration of canonical signaling may thus represent an important axis of therapeutics that is currently underexplored.

## Data availability statement

The original contributions presented in the study are included in the article/Supplementary material, further inquiries can be directed to the corresponding author.

## Author contributions

GV: Conceptualization, Funding acquisition, Investigation, Supervision, Writing – original draft, Writing – review & editing. V-ST: Writing – original draft. AL: Writing – original draft. TM: Funding acquisition, Writing – original draft. GE: Conceptualization, Methodology, Validation, Writing – original draft, Writing – review & editing. GH: Writing – review & editing.
