# Supplementary material for: Type I interferon signaling, cognition and neurodegeneration following COVID-19: update on a mechanistic pathogenetic model with implications for Alzheimer’s disease
Source: Front Hum Neurosci. 2024 Mar 18;18:1352118. doi: 10.3389/fnhum.2024.1352118 (PMC10982434; doi:10.3389/fnhum.2024.1352118)
Supplement: Supplementary file 1 [file Data_Sheet_1.docx]

# Supplementary Material 1. Type I interferon signalling dysregulation in the Alzheimer’s disease pathogenesis vs. COVID-19

In Alzheimer’s disease (AD), type I interferon signalling perturbations have been identified in the setting of microgliosis and several mechanisms that implicated several of the cellular and molecular mechanisms that drive its pathogenesis: microgliosis, beta amyloid (Αβ) accumulation and tau hyperphosphorylation. AD presents with irreversible and progressive deficits in memory and various cognitive and executive domains and consists one of the most formidable challenges in the field of neurodegenerative disorders. Comprising 60-80% of all dementia patients, AD stands as the predominant cause of dementia worldwide(1). Extracellular deposition of beta-amyloid plaques and intracellular neurofibrillary tangles (NFTs) consisted of hyperphosphorylated tau protein are the hallmark neuropathological features of the disease(2), eventually leading to neuronal death and gliosis (3).

The pathogenesis of AD is not clearly understood, but several factors, such as genetics, aging, lifestyle and environment (4), have shown a protein-conformational effect (3). In disease states, the normally soluble beta-amyloid, that derives from the cleavage of amyloid precursor protein (APP) by beta- and gamma-secretases, gives its place to insoluble, neurotoxic beta-amyloid oligomers (5). Polymerization of beta-amyloid oligomers into beta-amyloid_40_ and beta-amyloid_42_ leads to plague formation in the neocortex that can progress to the other structures like the midbrain, brain stem and cerebellar cortex (2). On the other hand, the accumulation of beta-amyloid causes hyperphosphorylation and subsequently oligomerization of the microtubule-associated protein tau results (MAPT) (6), a protein with a pivotal role in the stabilization and interconnection of microtubules. Thus, inter-neuron communication and signal propagation is hindered, and eventually neuronal apoptosis is promoted (7).

Notably, both beta-amyloid plagues and NFTs trigger an immune response of the innate immune system against protein conformation, with activation of microglial Toll-like receptors and subsequent recruitment of CNS-residing microglia(8). The inflammatory response, although beneficial in the initial phases of the disease thanks to the elimination of the pathologic aggregates and the secretion of neurotrophic factors (9), becomes harmful in the later phases of the disease, due to the secretion of considerable amounts of proinflammatory cytokines and chemokines that hasten neural decay (10). Additionally, tau-promoted senescence results in the release of insoluable NFTs from damaged microglia, that has been recently connected with cytotoxic T-cell responses, as shown in murine studies of tauopathy (11). Neurotoxicity becomes more and more evident as the pathology spreads and as the inflammation-associated increase in beta-amyloid NFTs accelerates.

A growing body of studies identify IFN-I responses as key molecular evens in the evolution of Alzheimer’s disease(12). In transgenic mice overexpressing interferon-stimulated genes beta-amyloid pathology has been evident (13). Experiments of microglia triggering with beta-amyloid and tau fibrils have resulted in IFN-I production, through mechanisms such as the facilitation of mitochondrial DNA release into the cytosol. It has been also demonstrated that inflammatory cytokines (especially IFN-I) make neurons susceptible to seeded tau aggregation (14). STAT3 phosphorylation around beta-amyloid plagues, elevation of IFNa levels, as well as IFNα and IFNβ mRNA upregulation was identified in an APPSWE/PS1ΔE9 mouse model of AD. IFN-I upregulation was also observed in other mouse models of AD, such as J20 mice, correlating with the noted behavioral changes (15). Recent research highlights the role of IFN-I in the pharmacotherapy of AD, noting that blockage of IFN signaling efficiently reduces tau aggregation in aged P301S-tau transgenic mice (14). Furthermore, both tau and beta-amyloid pathology have been shown to converge in cyclic GMP–AMP synthase (cGAS) – stimulator of interferon (IFN) genes (STING (STING) pathway, which links double stranded DNA sensing with IFN-I stimulation (16, 17),

**References**

1. Mahaman YAR, Embaye KS, Huang F, Li L, Zhu F, Wang JZ, et al. Biomarkers used in Alzheimer's disease diagnosis, treatment, and prevention. Ageing Res Rev. 2022;74:101544.

2. Goedert M. NEURODEGENERATION. Alzheimer's and Parkinson's diseases: The prion concept in relation to assembled Aβ, tau, and α-synuclein. Science. 2015;349(6248):1255555.

3. Tran L, Ha-Duong T. Exploring the Alzheimer amyloid-β peptide conformational ensemble: A review of molecular dynamics approaches. Peptides. 2015;69:86-91.

4. Adav SS, Sze SK. Insight of brain degenerative protein modifications in the pathology of neurodegeneration and dementia by proteomic profiling. Mol Brain. 2016;9(1):92.

5. Haass C, Selkoe DJ. Soluble protein oligomers in neurodegeneration: lessons from the Alzheimer's amyloid β-peptide. Nature Reviews Molecular Cell Biology. 2007;8(2):101-12.

6. Eftekharzadeh B, Daigle JG, Kapinos LE, Coyne A, Schiantarelli J, Carlomagno Y, et al. Tau Protein Disrupts Nucleocytoplasmic Transport in Alzheimer's Disease. Neuron. 2018;99(5):925-40.e7.

7. Combs B, Mueller RL, Morfini G, Brady ST, Kanaan NM. Tau and Axonal Transport Misregulation in Tauopathies. Adv Exp Med Biol. 2019;1184:81-95.

8. Heneka MT, Carson MJ, El Khoury J, Landreth GE, Brosseron F, Feinstein DL, et al. Neuroinflammation in Alzheimer's disease. Lancet Neurol. 2015;14(4):388-405.

9. Sarlus H, Heneka MT. Microglia in Alzheimer's disease. J Clin Invest. 2017;127(9):3240-9.

10. Aikawa T, Holm ML, Kanekiyo T. ABCA7 and Pathogenic Pathways of Alzheimer's Disease. Brain Sci. 2018;8(2).

11. Chen X, Firulyova M, Manis M, Herz J, Smirnov I, Aladyeva E, et al. Microglia-mediated T cell infiltration drives neurodegeneration in tauopathy. Nature. 2023;615(7953):668-77.

12. Sanford SAI, McEwan WA. Type-I Interferons in Alzheimer's Disease and Other Tauopathies. Frontiers in Cellular Neuroscience. 2022;16.

13. Roy ER, Wang B, Wan YW, Chiu G, Cole A, Yin Z, et al. Type I interferon response drives neuroinflammation and synapse loss in Alzheimer disease. J Clin Invest. 2020;130(4):1912-30.

14. Sanford SAI, Miller LVC, Vaysburd M, Keeling S, Tuck BJ, Clark J, et al. The type-I interferon response potentiates seeded tau aggregation and exacerbates tau pathology. Alzheimers Dement. 2023.

15. Taylor JM, Minter MR, Newman AG, Zhang M, Adlard PA, Crack PJ. Type-1 interferon signaling mediates neuro-inflammatory events in models of Alzheimer's disease. Neurobiol Aging. 2014;35(5):1012-23.

16. Govindarajulu M, Ramesh S, Beasley M, Lynn G, Wallace C, Labeau S, et al. Role of cGAS-Sting Signaling in Alzheimer's Disease. Int J Mol Sci. 2023;24(9).

17. Udeochu JC, Amin S, Huang Y, Fan L, Torres ERS, Carling GK, et al. Tau activation of microglial cGAS–IFN reduces MEF2C-mediated cognitive resilience. Nature Neuroscience. 2023;26(5):737-50.
