# Supplementary material for: Type I interferon signaling, cognition and neurodegeneration following COVID-19: update on a mechanistic pathogenetic model with implications for Alzheimer’s disease
Source: Front Hum Neurosci. 2024 Mar 18;18:1352118. doi: 10.3389/fnhum.2024.1352118 (PMC10982434; doi:10.3389/fnhum.2024.1352118)
Supplement: Supplementary file 2 [file Data_Sheet_2.docx]

# Supplementary Material 2. Limitations, strengths, and context

The interpretation of our model of cognitive impairment as well as the contribution of dysregulated tonic IFN-I and the crosstalk between peripheral and central nervous system immunity requires context and an awareness of important limitations.

We do not presume that COVID-19/PASC and Alzheimer’s disease are identical entities, but we stand to gain by studying mechanisms that are shared, such as Type I interferon perturbations.

Hence, an important consideration here is that observed cognitive impairment is not mechanistic evidence of this mechanism at work, but the sum of possible mechanisms, as well as other undiagnosed conditions (i.e., undiagnosed mild cognitive impairment or dementia)(108).

It is equally important to note however, that several confounders towards the establishment of causality between COVID-19 and cognitive impairment may feedback into IFN-I. Conditions such as aging, concurrent neurodegenerative disease, and structural damage induced by hypoxia and microvascular insult may contribute to cognitive impairment in the setting of COVID-19.(15, 108-110) Lung function and cognition specifically appear to be intertwined beyond infectious disease(111), mendelian randomization studies in chronic obstructive pulmonary disease (COPD) specifically however have suggested that this relationship may reflect shared risk factors rather than direct causality.(112) In a similar fashion, a substrate of vascular cognitive impairment (VCI) (113) in subjects subsequently infected with SARS-CoV-2 represents a reasonable confounder particularly when baseline neuroimaging and neurocognitive testing is not available, and that these two conditions share risk factors and pathogenic feedback(113, 114). As such, establishing causality, or contribution of COVID-19 to VCI cannot be safely and reasonably established when neuroimaging supports the latter.

In the setting of our model however, these conditions also contribute to a global dysregulation of the homeostatic IFN-I signalling in the CNS. As a case in point, ageing has been implicated in aberrant IFN-I responses in the choroid plexus resulting in cognitive impairment,(59) fully reflecting the effects of SARS-CoV-2 on the choroid plexus and cognition, albeit in a different timescale.(101) Similarly, both hypoxia and ischemia individually alter constitutional IFN-I in the brain,(109, 115, 116) and may thus contribute to differing levels of central nervous system interferonopathy.

Several other models of cognitive impairment have been reported in the literature and are not extensively reviewed in this perspective **(**see **Table 1** for a non-exhaustive list of recent theoretical models**)**. As cognitive impairment is a multifactorial phenomenon, it is likely that there is no single correct model, but rather a contribution of several factors that become evident as cognitive impairment and associated neuronal injury. In this context, our own model deals with several inevitable aspects of SARS-CoV-2’s encounter with our immune system in IFN-I, and correspondingly, the mechanistic consequences of its disruption. As such, whether a major or minor contributor in each case, our IFN-I centric model can explain specific aspects of cognitive impairment secondary to COVID-19, and how its pathogenesis may interact with Alzheimer’s.

The main advantage of studying IFN-I as a potentially causative mechanism for cognitive impairment as a manifestation of both long COVID-19 and Alzheimer’s disease, is that it is readily druggable. Studies in the pre- and post- pandemic era have considered a link between viral infection and Alzheimer’s disease pathogenesis, and a potentially inverse relationship with vaccination including a recent study by the National Institutes of Health (NIH) biobanks.(50) COVID-19 has been a focus of intense study and equally intense generation of multi-level data that have clearly outlined clinical, radiological neuropathological and genetic overlap with Alzheimer’s disease (reviewed in (3, 4, 23, 51, 52)).

**Table 1. Published models of cognitive dysfunction in long COVID**

| **Authors** | **Proposed model of cognitive dysfunction in long COVID** |
| --- | --- |
| Fontes-Dantas et al., 2023(102) | Authors support that SARS-CoV-2 S protein activates the Toll-like receptor 4 (TLR4), causing neuroinflammation and microglial phagocytosis of synaptic proteins, leading to memory and cognitive dysfunction. |
| Soung et al., 2022(103) | Authors support that SARS-CoV-2 leads to BBB disruption via cytokines, including IL-1β, which in turn permit entrance of cytokines and immune cells into the brain leading to activation of glial cells and impaired hippocampal neurogenesis contributing to cognitive and emotional symptoms. |
| Fernández-Castañeda et al., 2022(104) | Authors support that respiratory infection with SARS-CoV-2 can result in profound neuroinflammation, including the presence of IFN-γ, IL6, tumour necrosis factor alpha (TNF-α), chemokine-expressing cluster 10 (CXCL10), CCL7, CCL2, CCL11, granulocyte-macrophage colony-stimulating factor (GMCSF), and B-cell activating factor belonging to the TNF family (BAFF). They support a positive correlation between plasma CCL11 and cognitive deficits. They also support an inverse association between neurogenesis and reactive microglia/macrophages in the hippocampus. |
| Suzzi et al., 2023(22) | Authors support an association between cognitive ability after severe infection with SARS-CoV-2 and the type I interferon (IFN) antiviral response in the choroid plexus epithelium. |
| Mavrikaki et al., 2021(64) | Authors support that SARS-CoV-2 induces the upregulation of TNF and type I/II interferons which are implicated in cognitive decline, even in the absence of SARS-CoV-2 neuroinvasion. |
| Tan et al., 2022(105) | Authors support that the persistence of IFN-I and IFN-II levels in the serum of COVID-19 survivors is associated with chronic inflammation long-COVID-19 symptoms. |
| Quan et al., 2023(106) | Authors support that overexpression of angiotensin converting enzyme 2 (ACE2) over neurons and glial cells where SARS-CoV-2 binds is associated with cognitive dysfunction. Upon invasion, SARS-CoV-2 regulates reactive astrogliosis, microglial activation, and the neuroinflammatory cascade. Hence, the increased BBB permeability due to inflammation follows the disrupted brain homeostasis and neuronal death, causing cognitive decline. |
| Saucier et al., 2023(107) | Authors support that direct invasion of SARS-CoV-2 through the olfactory tract can lead to inhibition deficits due to local neuronal loss of frontal cortex. |
| Kurki et al., 2021(108) | Authors support that apoliporotein E ε4 allele (APOE4) is a risk factor for post-COVID-19 mental fatigue, which could be associated with increased cerebrovascular damage. |
| Abbrevations: ACE2: angiotensin converting enzyme 2; APOE4: apoliporotein E ε4 allele; BAFF: B-cell activating factor belonging to the tumour necrosis factor family; CXCL: chemokine-expressing cluster; GMCSF: granulocyte-macrophage colony-stimulating factor; IFITM: interferon-inducible transmembrane; INF: interferon; TLR4: Toll-like receptor 4; TNF-a: tumour necrosis factor alpha; | |
