# Supplementary material for: Type I interferon signaling, cognition and neurodegeneration following COVID-19: update on a mechanistic pathogenetic model with implications for Alzheimer’s disease
Source: Front Hum Neurosci. 2024 Mar 18;18:1352118. doi: 10.3389/fnhum.2024.1352118 (PMC10982434; doi:10.3389/fnhum.2024.1352118)
Supplement: Supplementary file 3 [file Data_Sheet_3.doc]

**Supplementary Figure 3. Dysregulation of peripheral tonic type I interferon signalling, its consequences in the CNS and a putative proteopathic-seed driven feed-forward circuit.** In COVID-19 associated cognitive impairment and Alzheimer’s disease, dysregulated type I interferon signalling has been detected both in the periphery and the central nervous system; disruptions in the microbiome populations in the airway, gut or oral cavity may mediate the shift from tonic IFN-I to its proinflammatory activation (See “1” in the figure). Multiple studies have shown that peripheral dysregulation of IFN-I is communicated to the CNS in a sterile manner (See “2” in the figure). In the hippocampi, dysregulation of IFN-I results in impaired neurogenesis and cognitive impairment (See “3” in the figure). Prolonged IFN-I dysregulation may lead to microgliosis, neuronal death and the induction of Αβ pathogenesis and tauopathy; the latter can be induced by IFN-I, and provide positive feedback, establishing a milieu of feed-forward IFN-I dysregulation.
