# Supplementary figures and images for: Type I interferon signaling, cognition and neurodegeneration following COVID-19: update on a mechanistic pathogenetic model with implications for Alzheimer’s disease
Source: Front Hum Neurosci. 2024 Mar 18;18:1352118. doi: 10.3389/fnhum.2024.1352118 (PMC10982434; doi:10.3389/fnhum.2024.1352118)

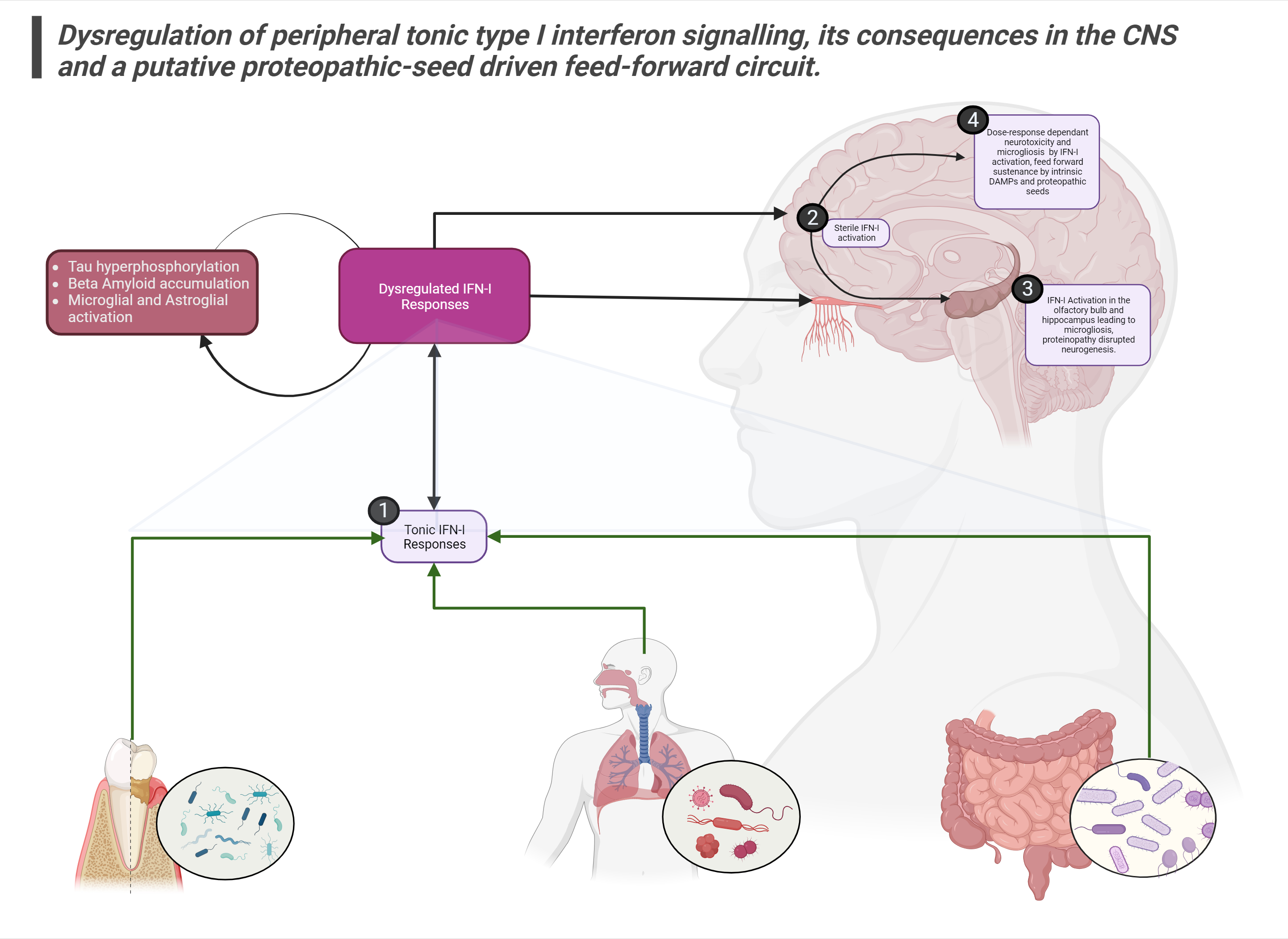

Supplement: Supplementary file 4 [file Image_1.JPEG]
